# Development of a Novel Microphysiological System for Peripheral Neurotoxicity Prediction Using Human iPSC-Derived Neurons with Morphological Deep Learning

**DOI:** 10.3390/toxics12110809

**Published:** 2024-11-11

**Authors:** Xiaobo Han, Naoki Matsuda, Makoto Yamanaka, Ikuro Suzuki

**Affiliations:** 1 Department of Electronics, Graduate School of Engineering, Tohoku Institute of Technology, 35-1 Yagiyama Kasumicho, Taihaku-ku, Sendai 982-8577, Japan; xiaobohan@tohtech.ac.jp (X.H.); na-matsuda@tohtech.ac.jp (N.M.); 2 Business Creation Division Organs on Chip Project, Usio Inc., 1-6-5 Marunouchi, Chiyoda-ku, Tokyo 100-8150, Japan; m.yamanaka@ushio.co.jp

**Keywords:** microphysiological system, human iPSC-derived sensory neuron, morphological deep learning, peripheral neuropathy

## Abstract

A microphysiological system (MPS) is an in vitro culture technology that reproduces the physiological microenvironment and functionality of humans and is expected to be applied for drug screening. In this study, we developed an MPS for the structured culture of human iPSC-derived sensory neurons and then predicted drug-induced neurotoxicity by morphological deep learning. Using human iPSC-derived sensory neurons, after the administration of representative anti-cancer drugs, the toxic effects on soma and axons were evaluated by an AI model with neurite images. Significant toxicity was detected in positive drugs and could be classified by different effects on soma or axons, suggesting that the current method provides an effective evaluation of chemotherapy-induced peripheral neuropathy. The results of neurofilament light chain expression changes in the MPS device also agreed with clinical reports. Therefore, the present MPS combined with morphological deep learning is a useful platform for in vitro peripheral neurotoxicity assessment.

## 1. Introduction

Novel therapeutics are necessary to treat neurological disorders such as Alzheimer’s disease, Parkinson’s disease, multiple sclerosis, and amyotrophic lateral sclerosis (ALS). Globally, in 2016, neurological disorders were the leading cause of disability-adjusted life years (276 million) and the second leading cause of death (9 million) [1]. Notably, 15% of children in the US aged 3 to 17 yr were affected by neurodevelopmental disorders [2]. However, there are not enough therapeutic options for neurological disorders, as most approaches merely address symptom alleviation [3,4]. To address the need for streamlining within drug development, more physiologically relevant models are needed to screen potential candidate compounds in nascent testing stages [5,6]. The overall goal should be to rule out cytotoxic drugs and drugs without human efficacy earlier, thus reducing the time and money spent on determining appropriate candidates. Understandings of neuronal functionality that can be studied using discrete, testable microphysiological systems (MPSs) are imperative to the development of therapeutics that move more towards regenerative therapies.

The advent of stem cell technologies and bioengineering in cell culture have led to the recent development of MPSs, which are expected to capture the human-like physiologies of tissues and organs in vitro, i.e., the structures and physiochemical factors of the tissues [7,8,9,10,11,12,13]. A neural MPS normally should be constructed with the proper cell sources, materials, and fabrication methods to recapitulate some organ-level functionality without requiring in situ or in vivo methods. In the present study, we developed an MPS device for culturing human iPS cell-derived neurons. This MPS device provides a structured neural culture that separates neurite grow-out into microfluidic channels. Human iPSC-derived sensory neurons were cultured in the MPS device. Then, the morphological changes in neurons under drug administrations were analyzed by a morphological deep learning method. After training AI models with images obtained from the MPS device, drug-induced neurotoxicity was detected for each compound.

## 2. Materials and Methods

### 2.1. MPS Device Fabrication

**Figure 1:**
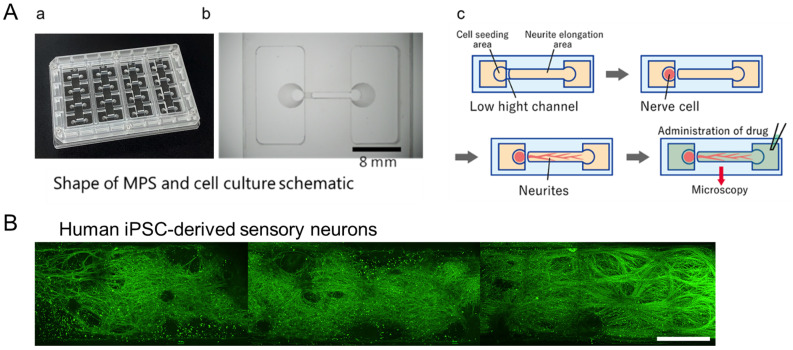
The microphysiological system (MPS) device. (**A**) MPS with (**a**) an overview. (**b**) Magnified view of a single channel. Scale bar = 8 mm. (**c**) Schematic of a series of test processes from culture to drug stimulation. (**B**) A representative immunofluorescence image of neurite elongation into the micro-channel in the MPS device. Scale bar = 500 µm.

Ushio Inc. prepared the MPS device as previously described [14]. Briefly, the vacuum ultraviolet (VUV) photobonding from an excimer light at a 172 nm wavelength was used to generate the functional groups (i.e., hydroxy and carboxyl groups) for assembling two Cyclic Olefin Polymer (COP) material layers directly with heat treatment. One MPS device is composed of four individual microfluidic cell culture channels (Figure 1A(a)). The middle narrow slot part of the channel is 1000 µm in width, 165 µm in length, and 40 µm in height, with an open-top channel (1000 µm in width, 6 mm in length) and two circular holes (2 mm in diameter) at both ends, which open to a rectangular medium reservoir (15 mm in width, 8 mm in length, and 5 mm in height). The maximum volume in each channel containing reservoirs is 1 mL. COP material (Zeonex 690R, Zeon, Tokyo, Japan) was injected into the two molds individually. The components were irradiated with VUV from an excimer lamp (172 nm; Ushio Inc., Tokyo, Japan) at 25 °C after taking the structured COP components from the molds. The component surfaces were assembled using a heat press at less than 132 °C. Finally, ethylene oxide gas (Japan Gas Co. Ltd., Kanagawa, Japan) was used for device decontamination.

### 2.2. Cell Culture

Before seeding human iPSC-derived sensory neurons, the surface of the MPS device was coated sequentially by Cellmatrix collagen type I-C solution (637-00773, Nitta Gelatin, Tokyo, Japan), Poly-D-lysine solution (P7405, Sigma-Aldrich, St. Louis, MO, U.S.), and SureBond-XF coating solution (ax0053, AXOL Bioscience, Easter Bush, U.K.), each for 1 h. All plating and maintenance media were obtained from Axol’s kit (ax0157, Axol Bioscience, Easter Bush, U.K.). Cryopreserved human iPSC-derived Sensory Neuron Progenitors (ax0055, Axol Bioscience) were thawed and suspended in the plating medium. For dispersed culture, approximately 5.0 × 10^4^ cells in a 15 µL neuron medium were seeded directly into the seeding chamber at one side of the MPS device. After 30 min, 600 µL of plating medium was applied to the whole MPS device. The total volume of the medium was replaced with maintenance medium containing growth factor (Sensory maturation maximizer supplement, GDNF: 25 ng/mL, ax139855, NGF: 25 ng/mL, ax139789, BDNF: 10 ng/mL, ax139800, NT-3: 10 ng/mL, ax139811) and maximizer (sensory maintenance medium) after 24 h. Following this, half of the medium was replaced every 3 days.

After 3 weeks (18~20 days) in culture, test drugs were administered into the cultures to evaluate cell responses. Considering the purpose of toxicity prediction, several anticancer drugs with different modes of action were used in the present study. The tested drugs included paclitaxel and vincristine, known inducers of axonal damage [14,15], as well as oxaliplatin, which causes soma damage [16]. For validation, bortezomib, a proteasome inhibitor associated with peripheral neuropathy [17], and suramin, an antiparasitic drug with antineoplastic effects but known to damage myelin [18], were chosen. The drugs’ effects on rodent primary peripheral neurons were detected using the MPS device as reported in our previous study [19]. Based on the neurotoxicity prediction results in our previous study [19], the dosage of test drugs was set in the present study as paclitaxel at 0.1 µM and 1 µM, vincristine at 0.003 µM and 0.03 µM, oxaliplatin at 10 µM and 100 µM, bortezomib at 0.01 µM, and suramin at 10 µM and 100 µM. Dimethyl sulfur oxide (DMSO, 0.1%) was added as the negative control drug to the cultures. The drug exposure lasted for 24 h at 37 °C.

### 2.3. Immunocytochemistry

Sample cultures were fixed with 4% paraformaldehyde in PBS on ice (4 °C) for 10 min. Fixed cells were incubated with 0.2% Triton-X-100 in PBS for 5 min, then with preblock buffer (0.05% Triton-X and 5% FBS in PBS) at 4 °C for 1 h, and, finally, with preblock buffer containing a specific primary antibody, mouse anti-β-tubulin III (1:1000, T8578, Sigma–Aldrich), at 4 °C for 24 h. Samples were then incubated with the secondary antibody, anti-mouse 488 Alexa Fluor (1:1000 in preblock buffer, A-11001, Invitrogen, Waltham, MA, U.S.), for 1 h at room temperature. Stained cultures were washed twice with preblock buffer and rinsed twice with PBS. An inverted microscope with a confocal imaging system (AX/R, Nikon, Tokyo, Japan) was used to visualize immunolabeling for local images and whole microchannel-length images. ImageJ software (Version 1.54k 15, NIH) was used to adjust the image intensity.

### 2.4. Deep Learning for Image Analysis and Toxicity-Positive Prediction

Image treatment and AI creation were performed using a similar method as described before [19]. Briefly, immunofluorescence images of neurites from neurite elongation areas were segmented into 576 × 576-pixel images and used for AI analysis. Our algorithms were developed using MATLAB (Version 2021b)’s Deep Learning Toolbox. To develop the image recognition model, we performed transfer learning of GoogLeNet using the “TrainNetwork” function from the Deep Learning Toolbox. Vincristine images were trained as neurite toxicity-positive and oxaliplatin as cytotoxicity-positive; then, all segmented images were tested, and the percentage that was determined to be cytotoxicity/neurite toxicity-positive was calculated as the toxicity probability.

### 2.5. ELISA Kit Assay

After compound exposure, the medium supernatant in each micro-channel was collected (N = 1 sample for each micro-channel, and N = 4 samples for each condition), and the expression of human neurofilament light chain (NF-L) was analyzed using a Human NF-L ELISA kit (KE00305, Proteintech, Rosemount, IL, U.S.) following the manufacturer’s protocol. Briefly, 100 µL of standard solution or supernatant sample was added to the appropriate well in a 96-well microplate. After 2 h incubation at 37 °C, the solution was removed, and the well was treated with antibody solution, HRP solution, and TMB Substrate, each for 1 h at 37 °C in sequence. Finally, Stop solution was added to each well, and the microplate was immediately readout at 450 nm using a multi-microplate reader (Infinite F PLEX, TECAN, Männedorf, Switzerland).

### 2.6. Statistical Analysis

For the results of NF-L expression by ELISA kit, a Student *t*-test was used to calculate the significant difference between each compound and the vehicle at different concentrations.

## 3. Results

### 3.1. Morphological Presentation of Human iPSC-Derived Sensory Neurons in the MPS Device

**Figure 2:**
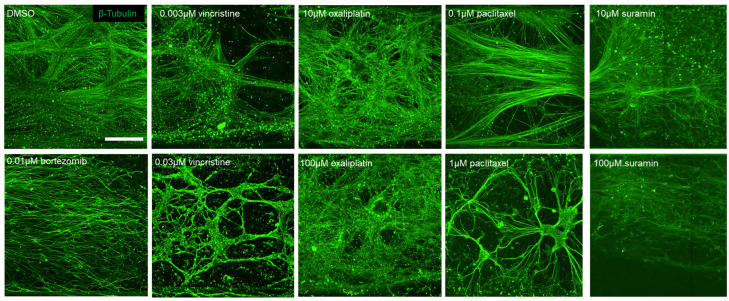
Representative β-tubulin immunofluorescence images in the MPS device after drug administration. Scale bar = 100 µm.

After 3 weeks of culture, human iPSC-derived sensory neurons showed sufficient neurite growth-out with unidirectional elongation into the microchannel (Figure 1B). Then, neurons were exposed to various anticancer drugs and, subsequently, 24 h post-exposure, immunostained images were captured. The tested drugs included paclitaxel and vincristine, known inducers of axonal damage, as well as oxaliplatin, which causes soma damage. DMSO was utilized as a negative control. For validation, bortezomib, a proteasome inhibitor associated with peripheral neuropathy, and suramin, an antiparasitic drug with antineoplastic effects but known to damage myelin, were chosen. Figure 2 shows the local immunofluorescence images of cultured human iPSC-derived sensory neurons at 3 weeks in the MPS device after drug administration. Morphological alterations in response to the compounds were evaluated within the micro-channel area. Under the negative control DMSO, unidirectional neurite elongation was observed throughout the microchannel. Under the administration of 0.003 µM vincristine, axonal degeneration was observed in the forms of fragment and hollowing out. Thus, the phenomenon was more clear under 0.03 µM vincristine. Under the administration of oxaliplatin at low and high concentrations, axonal aggregation was observed. A clear depletion in axons was observed under the administration of paclitaxel, suramin, and bortezomib.

### 3.2. Neurotoxicity Prediction for Human iPSC-Derived Sensory Neuron Using Morphological Deep Learning

**Figure 3:**
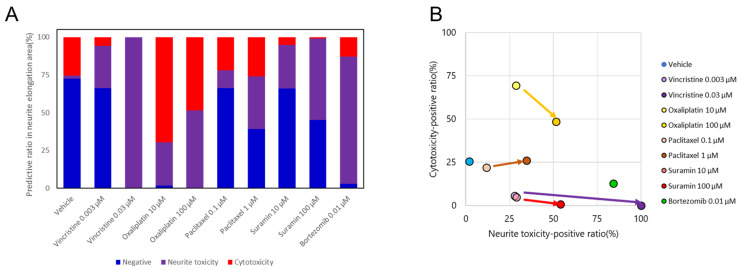
Prediction results of selected compounds by morphological deep learning analysis. (**A**) Distribution of the predicted ratio as negative, positive for neurite toxicity, or positive for cytotoxicity. (**B**) Classification of selected compounds based on the prediction results. The predicted probability of each compound was plotted with cytotoxicity as the vertical axis and neurite toxicity as the horizontal axis.

In order to predict neurotoxicity and the influence point (i.e., cytotoxicity or neurite toxicity), an AI was created by training with DMSO image datasets as negative, oxaliplatin images as cytotoxicity, vincristine images as neurite toxicity, as described above. The predictive neurotoxicity-positive ratio for each compound is shown in Figure 3. DMSO was detected as negative with a high negative ratio. For vincristine, the neurite toxicity-positive ratio showed a significant increase and reached a maximum at a high concentration. Oxaliplatin was predominantly detected as cytotoxicity-positive but with an increase in the neurite toxicity-positive ratio at high concentrations. A dose-dependent increase in the neurite toxicity-positive ratio was observed under the administration of paclitaxel and suramin. Bortezomib was predicted as neurite toxicity with a high positive rate. The outcomes for each compound were plotted to examine their distributions with the cytotoxicity-positive ratio as the vertical axis and the neurite toxicity ratio as the horizontal axis (Figure 3B). Oxaliplatin exhibited a shift in the y-axis direction, while vincristine primarily moved in the x-axis direction. Paclitaxel shifted in the upper-right quadrant, indicating a simultaneous increase in both axonopathy and cytotoxicity. Suramin also exhibited a dose-dependent shift along the x-axis, similar to that of vincristine. Bortezomib positioned itself close to the vincristine position at a high concentration.

### 3.3. Expression Change in NF-L After Drug Administration

**Figure 4:**
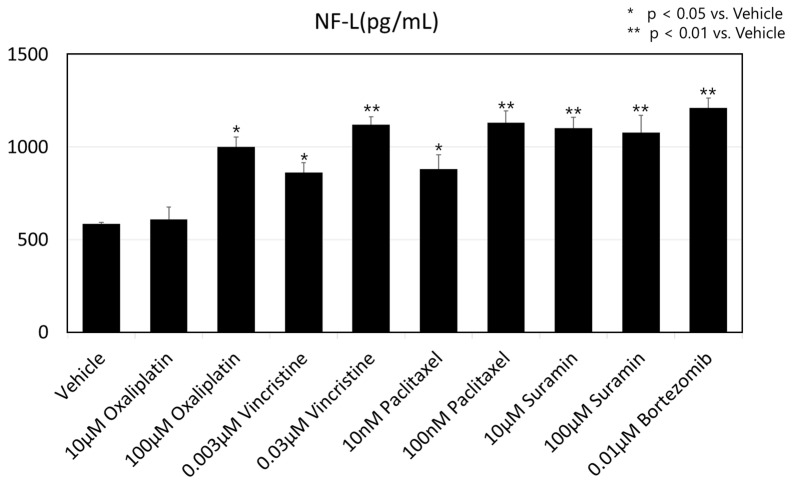
Expression value of human neurofilament light chain (NF-L) from medium supernatant after drug administration. The results are shown as mean + SD, * *p* < 0.05 vs. vehicle (DMSO), ** *p* < 0.01 vs. vehicle.

Figure 4 shows the NF-L expression in medium supernatant after compound administration with a Human NF-L ELISA kit, as described above. Administration of all anti-cancer drugs induced an increase in NF-L expression. A significant difference vs. vehicle was detected for all conditions except 10 µM oxaliplatin.

## 4. Discussion

Culturing of neuronal and glial cells has been extensively reviewed previously [20,21]. While many of these culture systems focus on non-human cultures, emerging protocols for the rapid generation of human nervous systems from iPSC are of particular interest to MPS development. In this study, we developed a COP-based MPS device that was proven to maintain human iPSC-derived neuron growth without undesired cellular damage. The microchannel structure ensured rapid neurite growth-out with unidirectional elongation. This strategy allows screening applications for human iPSC-derived sensory neurons at 3 weeks of culture, which provides the potential to investigate human-reliable pathological factors.

Several anti-cancer drugs used in cancer treatment could induce chemotherapy-induced peripheral neuropathy (CIPN) as an adverse effect [22,23]. In the present study, we attempted to quantify drug-induced neuron degenerations using MPS cultures with the deep learning analysis method. The results showed that the influence of several representative anticancer drugs was successfully detected with human iPSC-derived sensory neurons. In the MPS device, the micro-channel structure allowed abundant neurite growth-out with unidirectional elongation. After training with image datasets from the micro-channel area, test compounds could be further separated as cytotoxicity or neurite toxicity. Previously, our group reported a similar approach to predict neurotoxicity induced by anti-cancer drugs using rodent primary sensory neurons cultured in the MPS device [19]. Compared to the previous result, the plot map of each compound showed the same distribution in the current study, which indicates the potential for valid drug screening beyond the species gap. However, the morphology of cell bodies was analyzed and trained to AI models in the previous research. The same treatment was not performed in the present study since the cell bodies of human iPSC-derived sensory neurons were much smaller in size and difficult to separate individually for deep learning. New approaches to cell culture and image treatment are now under development to enable analysis including cell bodies for a more accurate prediction.

The functionality of a neuronal system is paramount for meaningful data acquisition. Recently, neurofilaments (NFs) have emerged as a promising biomarker for peripheral neurological disorders, with ongoing studies assessing the promising performances of NFs for both disease diagnostics and prognosis [24,25,26]. NFs, especially the light chain (NF-L) and the phosphorylated heavy chain (pNF-H), were detected in the peripheral blood of ALS patients [27,28]. In the present study, we confirmed the increase in NF-L expression in medium supernatant after the administration of anti-cancer drugs using the MPS device. This result agreed with several previous clinical studies. For example, an increase in the serum NF-L level was observed in oxaliplatin-induced peripheral neuropathy patients [29]. After treatment with vincristine, paclitaxel, or bortezomib, an acute high blood level of NF-L with continuous elevation was observed in patients who were experiencing symptoms of CIPN [30,31,32]. Therefore, the current MPS device provides possible clinical translation for CIPN with NF-L as a biomarker. By further analysis of the relationship between NF-L expression and neurite morphology, it is possible to elucidate the functional role of NF-L in axonal damage that is not clear yet.

Taken together, the current MPS device shows the potential for in vitro assessment of peripheral neuropathy. Using human iPSC-derived sensory neurons, drug-induced toxicity could be detected at low concentrations with a morphological deep learning method. And the increase in NF-L expression indicates possible clinical translation.

## Data Availability

The original contributions presented in this study are included in this article. Further inquiries can be directed to the corresponding author.
